# Impact of 360° vs 2D Videos on Engagement in Anatomy Education

**DOI:** 10.7759/cureus.14260

**Published:** 2021-04-02

**Authors:** Vivian Chan, Nathaniel D Larson, David A Moody, David G Moyer, Neeral L Shah

**Affiliations:** 1 Internal Medicine, University of Virginia School of Medicine, Charlottesville, USA; 2 Medical Education, University of Virginia School of Medicine, Charlottesville, USA; 3 Gastroenterology, University of Virginia, Charlottesville, USA

**Keywords:** medical education, anatomy, virtual reality, online learning, engagement, 360 video, technology

## Abstract

Medical education is constantly evolving, especially as students were forced to study from home during the coronavirus disease 2019 (COVID-19) pandemic, and new technologies have driven the rapid development of supplemental online educational resources. In this study, we examine if 360° videos can promote increased engagement over standard two-dimensional (2D) videos among medical students learning anatomy. We enrolled 39 fourth-year medical students to watch two four-minute videos of anatomy lab exercises in a 360° three-dimensional format using an immersive headset or in a 2D format on a laptop computer. Every two minutes, students were asked to rate their engagement from 0-100. Following the videos, they reported their degree of agreement with 14 statements related to engagement, practicality, and interest in the technology. While watching the videos, the average engagement reported by the 360° video group was higher at each time point than the engagement reported by the two-dimensional group. Further, the engagement remained high in the 360° group through the six- and eight-minute timepoints. In the post-video survey, the 360° group reported a statistically significantly higher average engagement in seven of eight measures on the assessment. A 360° video was rated as more practical and interesting than a two-dimensional video. No significant difference existed in the perceived ease of learning. Overall, the use of 360° video may improve engagement for short videos used in medical education. However, developing a better understanding of its impact on learning outcomes will be critical for determining the overall value and effectiveness of this tool.

## Introduction

In the current climate of online learning, it is unclear if the technology that is being used in medical education is effective in engaging students more than the traditional approaches used in the classroom. Substantial curricular change has occurred at schools across the United States and specific trends have helped accelerate this curricular innovation, including the “new science” of learning, technology, and instructional methods [1]. Technology in medical education has seen explosive growth, as infection risk from the pandemic continues to limit face-to-face interaction and has forced educators to find new methods of teaching [2]. As we investigate these new tools for learning, it is important that we understand whether learners will engage with the use of this technology.

A literature search for publications featuring “online learning” on PubMed yields 456 results published in the year 2020. Generations who have been surrounded by modern technology through their entire lifespan are typically comfortable with technology, process this information very differently than preceding students, and overall have high proficiency in web-based, self-directed learning and media literacy [3-4]. As such, new forms of “e-learning,” or learning utilizing electronic technologies, such as video conferencing, podcasts, virtual simulations, teleconferences, online resources, and interactive media, are well-received, easily accessible methods of providing students with more personalized control over the pace and content of their learning [2-6]. The use of online curricula is not new, and Draus et al. demonstrate that with the addition of instructor-generated video content, students report higher engagement and overall satisfaction in an asynchronous online course [7].

Before the coronavirus disease 2019 (COVID-19) pandemic, we had implemented the use of modern imaging and diagnostic techniques as part of an innovative, integrative anatomy laboratory experience for pre-clerkship students. This exercise was designed to enhance clinical relevance and help students go beyond rote identification and memorization of anatomic structures [8]. However, in the midst of the pandemic, like other medical schools, we have shifted to online learning in order to adhere to social distancing guidelines and to decrease the risk of infection. More specifically, for the pre-clerkship curriculum, there was a need to address additional barriers related to group-based instruction and the dissection of cadavers in the anatomy lab. Medical schools have worked to transition content/resources for learning to an online format, including self-study materials, video podcasts, video conferencing, small-group virtual settings, and discussion/email forums [9].

With the COVID-19 pandemic, we explored novel ways to adapt the integrative laboratory experience model. The recent advent of augmented reality (AR), virtual reality (VR), and 360° video has provided opportunities to expand methods of e-learning, and they have started to appear in the realm of medical training. In AR, a user is presented with a computer-generated image superimposed onto a part of normal reality [10]. In VR, a user is exposed to a computer-generated environment completely separated from normal reality [11]. Similar to these modalities, a 360° video allows users to experience an immersive environment, though, unlike AR and VR, they are unable to move within or interact directly with objects in the virtual environment. These augmented and virtual experiences have been shown to increase the technical accuracy of residents prior to performing their first colonoscopy or endoscopy, increase the suturing speed of surgeons, and improve the recognition of anatomical landmarks [12]. What is unclear is if student engagement is maintained or improved through the use of these modalities. The aim of our study was to compare a 360° video to a two-dimensional (2D) desktop video and its ability to promote the engagement of students in a clinical anatomy and imaging integrative laboratory experience.

## Materials and methods

Materials and methods

The educational videos used in this study were recorded during the previously implemented pre-clerkship clinical anatomy and imaging laboratory (CAIL) [8]. This lab is taught at the end of the gastrointestinal organ system and incorporates foundational pre-clerkship material as clinical procedures are demonstrated on cadavers in the anatomy lab. During this lab, we recorded two of the stations for playback in a 360° video and traditional 2D video: the liver biopsy station on a formaldehyde-preserved cadaver and an upper endoscopy station on a soft-embalmed preserved cadaver.

Technical equipment

The videos were captured with a Samsung 360° Round Stereoscopic 3D camera (Samsung Group, Seoul, South Korea) and a NeXT PC stitching computer (NeXT Inc., Fremont, California). The camera was used to record the upper endoscopy and liver biopsy demonstration videos during the gastrointestinal clinical anatomy lab for first-year medical students.

Camera hardware and rigging

During the anatomy lab exercises, a Samsung 360° Round SM-R260 was boomed 7 feet and inverted over the procedure table at the liver biopsy and endoscopy stations to avoid disrupting the learners participating in the activity. The camera has 17 4k lenses consisting of an eight-pair configuration to allow for stereoscopic rendering and a single top-side lens with six spatially arranged microphones along the edge of the camera. This camera streamed live videos to the stitching computer.

Video processing and editing

The video was edited for sound leveling and lighting adjustment using Adobe Premiere (Adobe Inc., Mountain View, California). The 360° video was rendered in monoscopic format. The 2D videos were static and cut to focus on the instructor and procedure table but ensured that both were in view for the entire lesson.

Viewing the video

The 360° video was uploaded directly to a 64 GB Oculus Go Headset (Oculus VR, Irvine, California) to allow direct viewing from the headset and associated hand controller without a wireless Internet connection. The 2D videos were uploaded onto a web-based playback platform and viewed on a 13-inch laptop.

Participants

Study participants were recruited from the fourth-year medical school class via e-mail. Students who had completed all pre-clerkship courses and core rotations were eligible. The decision to recruit these students was based on the desire to have a study population consisting of individuals at the same level of training who had not recently participated in the anatomy lab learning experiences recorded for this study; only fourth-year medical students met these criteria. This decision was meant to minimize confounding variables that may affect student engagement.

Participation was voluntary, and all participants signed informed written consent.

Study design

The study recruitment and data collection were completed over a period of 60 days. Participants were randomized into two groups in a 1:1 ratio by recruitment number: Group A viewed the 360° videos using the Oculus Go VR headset while Group B viewed the 2D videos on a laptop. Each group watched both a four-minute demonstration and explanation of upper endoscopy as well as a four-minute demonstration and explanation of liver biopsy in the same order.

Halfway through and at the end of each video, participants were asked two questions to gauge their engagement (at the 2, 4, and 6 min mark): (1) Are you thinking any thoughts or images unrelated to the video?; (2) How engaged are you on a scale from 0-100? Participants were made aware that these questions would be asked before beginning to watch the videos and were instructed not to pause the videos when the questions were asked.

After watching the second video, participants completed a Google Forms survey (Appendix) to assess their attitudes toward and familiarity with new technology and their engagement and interest in the videos presented. The survey was adapted from a user experience questionnaire that has been used in gaming literature involving the same Oculus Headset used in the present study [13]. The questions were chosen specifically to sample specific subscales defined by the original survey (engagement, immersion, flow, skill, emotion, and judgment). Participants answered background questions to establish a baseline for their knowledge and attitudes towards new technologies, including whether they had prior experience with 360° videos. Participants then ranked from a score of 0-100, with 100 being in complete agreement, their agreement with statements assessing engagement, interest in technology, ease of use, ease of learning, and practicality in regards to the respective video medium. Following the survey, participants were given the option to view the same videos using the other modality.

The study was approved by the University of Virginia Institutional Review Board for the Social and Behavioral Sciences (IRB-SBS).

Statistical analysis

The arcsine transformation was applied to the proportion, engagement score/100, where engagement was scored from 0-100 (100 = complete engagement). The transformed scores served as the outcome variable in a repeated measures model and contained the following independent categorical variables: time (2 min, 4 min, 6 min, 8 min), group (2D, 360°), and all possible interaction terms. The Wilcoxon rank-sum test and the chi-square test of association were used to assess responses to the post-study survey.

## Results

Thirty-nine (39) fourth-year medical students participated in the study. The 360° group had 20 participants, and the 2D video group had 19 participants. There was no statistical difference between the two groups in regards to previous experience with 360° video. While watching the videos, the average engagement reported by the 360° group was higher at each time point than the engagement reported by the 2D group (Table 1).

**Table 1:** 2D and 360° video mean engagement scores between two to eight minutes

	2D Video			360 Video			
Time (min)	N	Mean	SD	N	Mean	SD	p-values
2	19	79.3	12.2	20	84.3	11.2	0.1479
4	19	74.6	13.9	20	82.0	11.5	0.0728
6	19	66.5	14.4	20	88.7	8.3	<0.0001
8	19	58.0	19.1	20	87.1	9.9	<0.0001

**Figure 1:**
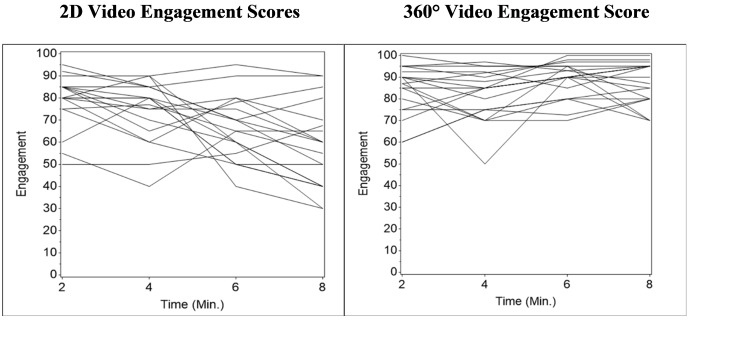
2D vs. 360° video engagement scores from two to eight minutes in playback

This difference was statistically significant at the six and eight-minute time points. Overall, engagement scores in the 2D group demonstrated a downward trend while those in the 360° group did not (Figure 1).

In the post-video survey, there were statistically significant differences (p<0.05) between the two groups in regards to engagement, stimulated learning, enjoyment, practicality, and interest in the technology (Table 2).

**Table 2:** Mean level of agreement with post-video survey statements among 2D and 360° video users Participants were asked to give a score from 0-100 based on how much they agreed with each of 14 statements (summarized in parentheses) with the score of 0 being in complete disagreement and the score of 100 being in complete agreement. Numbers displayed are the means for each question for each group. Comparisons were made with the Wilcoxon test. For the full list of questions, see the Appendix.

	2D	360°	p-value		2D	360°	p-value
Q1 (“compelling”)	71	86	0.002	Q8 (“enjoyed”)	55	94	<0.001
Q2 (“felt involved”)	29	80	<0.001	Q9 (“mind wandered”)	63	46	0.160
Q3 (“stimulated”)	40	84	<0.001	Q10 (“can use the tech”)	100	85	<0.001
Q4 (“honed in”)	27	76	<0.001	Q11 (“practical”)	53	77	0.007
Q5 (“lost sense of time”)	31	74	<0.001	Q12 (“exciting - tech”)	38	91	<0.001
Q6 (“exciting - videos”)	27	90	<0.001	Q13 (“easy to learn”)	73	86	0.450
Q7 (“easy to use”)	99	95	0.024	Q14 (“more interesting”)	57	94	<0.001

Seven out of eight questions assessing engagement were rated significantly higher in the 360° video group as opposed to the 2D group (Q1-6 and Q8 p < 0.05; Q9 p = 0.16), including feeling more stimulated and involved in the lab experience. Although the 2D group reported more ease and confidence with using the video playback feature (Q7 and Q10 p < 0.05), there was no statistical difference in regards to perceived ease of learning between the two groups (Q13 p = 0.45). The 360° video was also rated as more practical (Q11 p = 0.007) and interesting (Q12 and Q 14, p < 0.001) than 2D.

## Discussion

Short 360° videos were more engaging for learners than 2D videos: seven out of the eight survey questions assessing these factors were rated higher in the 360° video group. More students in the 360° video group report feeling more engaged or involved than those in the 2D group. Neither modality was difficult to use and the participant’s focus does not vary greatly, yet overall engagement remained higher in the 360° videos.

These results support previous findings by Harrington et al. [14] who conducted a similar study related to 10-minute, 360° surgical videos. Shorter videos have been shown to more effectively maintain student engagement, with a median engagement time of at most six minutes despite differences in the total video length [15]. In our study, the engagement of the 360° video group remained high, even at the six- and 8-minute time points, in contrast to the decline of engagement of the 2D group as the videos progressed. However, the switch between the two videos at the four-minute time point may have contributed to maintaining engagement.

In addition to maintaining engagement, shorter videos may also be advantageous for users who experience the side effects of nausea, headache, and eye strain associated with a 360° video, although we did not encounter nor specifically assess for these symptoms in our study [16]. Additionally, the 360° video was rated as more interesting and practical than 2D. It remains unclear if this increased interest would be maintained if the students were to also increase their familiarity with the technology as most students denied prior 360° video or VR usage. Familiarity with the content of the videos, however, serves as a potential limitation to the study. Although the fourth-year medical students had never participated in the CAIL exercise, they may have been exposed to upper endoscopy and liver biopsies in their clinical rotations. The use of content that is new to learners could lead to further differences in engagement levels. For example, it may be helpful to explore if engagement differs in future studies with pre-clerkship students being introduced to new curricular materials through 2D videos versus 360° videos.

A lesson learned in creating the videos was the need to produce videos in an isolated environment with video learning in mind, rather than recorded directly from an unfiltered feed to improve sound quality and avoid background noise. Using resources in this way may allow 360° videos to serve as effective, alternative forms of learning in the absence of face-to-face teaching, as students would be able to replay content and learn at their own individual pace within a stimulating environment while limiting contact with others. The technology in this study utilized highly specialized equipment and video processing software; however, with the increased demand for e-learning, and the landscape of this modality constantly evolving, more affordable and practical equipment could be used to produce these videos and converted on personal computers.

A recent meta-analysis comparing online to offline learning in undergraduate medical education suggested that online learning, including computer-based modules, virtual lectures, and recorded videos, is at least as good as - or possibly better than - offline learning when students were assessed on both knowledge and skill development [17]. With this in mind, determining the overall value of designing innovative 360° videos for medical curricula will depend on the extent to which this new modality can improve outcomes already seen in other forms of online learning. Of note, increased engagement has been shown to allow students to feel more stimulated and to make them more likely to learn [18]. Additionally, other immersive modalities, such as AR and VR, have already demonstrated great potential as supplemental learning tools for medical education [19]. As disruptions in face-to-face learning continue to impact learners, these emergent technologies may help us continue to adapt to a new reality [20].

Limitations

Although we noted increased engagement and positive feedback associated with a 360° video, a major limitation of our study is that we did not evaluate knowledge gain or retention. Therefore, it is difficult to draw any conclusions comparing the effectiveness of the use of these technologies to enhance knowledge. Future studies should assess retention using videos introducing new concepts and assess changes in knowledge as a result of the use of these technologies.

## Conclusions

Overall, the use of a 360° video in medical education continues to show promise as an effective learning tool. Though knowledge retention was not specifically tested for, it is reasonable that the increased student engagement seen with the use of 360° videos may contribute to improved learning outcomes. This is especially true during a pandemic that has made face-to-face learning more difficult and that has forced students and teachers to embrace more virtual educational tools. However, future studies will need to assess how 360° video impacts knowledge retention in order to fully understand the utility of this technology in promoting learning.

## Appendices

List of survey questions from Table 2

On a scale of 0-100, indicate how much you agree with the following statements.

For example:

I attend the University of Virginia - 100% agree

Clouds in the sky are red - 0% agree

Q1. The instructors in the video were compelling. (subscale - engagement)

Q2.I felt involved in the lab experience from the video. (subscale - engagement)

Q3.I felt stimulated by the video of the lab exercise. (subscale - immersion)

Q4.I was so involved in the video that I was not aware of things happening around me. (subscale - immersion)

Q5. I lost my sense of time watching the video. (subscale - flow)

Q6.I felt watching the video was an exciting experience. (subscale - flow)

Q7. I thought the video playback was easy to use. (subscale - skill)

Q8.I enjoyed the video experience presented to me. (subscale - emotion)

Q9.I thought my mind wandered during the video experience. (subscale - emotion)

Q10.I felt confident using the controls to playback the video. (subscale - skill)

Q11.Learning from this video format for class is practical. (subscale - judgment)

Q12.Learning from this video format is exciting. (subscale - judgment)

Q13. Learning from this video format is easy. (subscale - judgment)

Q14. Learning from these types of videos would make studying more interesting. (subscale - judgment)
